# When the user is not the chooser: stakeholder involvement in innovation adoption and implementation for addressing HCAIS

**DOI:** 10.1186/1753-6561-5-S6-O36

**Published:** 2011-06-29

**Authors:** R Ahmad, Y Kyratsis, A Holmes

**Affiliations:** 1 Infectious diseases and Immunity, Imperial College London, London, UK

## Introduction / objectives

Whilst evidence based innovations exist for helping to address Health Care Associated Infections (HCAIs), the uptake and implementation of these is highly variable and in some cases very slow. We aimed to investigate organisational innovation adoption decisions and implementation processes in the context of Infection Prevention and Control (IPC). Here we focus on the implications of stakeholder involvement during these processes.

## Methods

We sampled NHS trusts in England, which were winners of the Department of Health 'HCAI Technology Innovation Award 2009'. By analysing data from over 100 semi-structured qualitative interviews with clinical and non-clinical staff at all levels, we looked at technology selection decisions and implementation processes.

## Results

Stakeholder involvement varied across the trusts with decisions highly exclusive to the IPC team, to highly inclusive of wider trust members. The context, including previous experience, and logistical factors influenced the level of stakeholder engagement. The method and timing of stakeholder involvement impacted on: the nature of innovations considered, innovations selected, success of the implementation of innovations. Cases of non-adoption and discontinued technologies are presented for important learning. Cases of successful implementation are presented in context of the adopting hospital. A model of potential benefits to ‘successful’ innovation adoption and implementation is presented.

## Conclusion

Key stakeholder involvement can lead to innovation adoption decisions compatible with structural and cultural contexts. There are potential synergies through stakeholder engagement across the two phases of decision making and implementation. Our model has useful application as a strategic and operational toolkit for IPC.

## Disclosure of interest

None declared.

